# Identity of the leafhopper *Kolla albescens*, with new synonymy (Hemiptera, Cicadellidae)

**DOI:** 10.3897/zookeys.420.6899

**Published:** 2014-06-25

**Authors:** Ze-hong Meng, Mao-fa Yang, Mick Webb

**Affiliations:** 1 Institute of Entomology, Guizhou University; Guizhou Provincial Key Laboratory for Agricultural Pest Management of the Mountainous Region,; 2 Guiyang Guizhou, 550025, P. R. China; 3 Guizhou Tea Research Institute, Guiyang Guizhou, 550006, P. R. China; 4 The Natural History Museum, London, UK.

**Keywords:** Auchenorrhyncha, China, new combination, Pagaroniini, taxonomy

## Abstract

Newly collected male and female specimens of the leafhopper *Kolla albescens* Jacobi, 1943 from the type-locality (Northeast China), are identified as *Pagaronia albescens* (Jacobi), **comb. n.** (Evacanthinae: Pagaroniini). A redescription of the species is provided together with habitus photographs of the male and female and illustrations of the male and female genitalia. *P. continentalis* Anufriev, 1970 is placed as a junior synonym of *P. albescens* **syn. n.**

## Introduction

The leafhopper *Kolla albescens* was described by Jacobi (1943) based on a female specimen from Northeast China. In Young’s (1986) Cicadellinae revision he did not examine the type specimen of *Kolla albescens* so placed it in *Kolla* following Metcalf (1965). Although Wilson et al. (2009a, b) provided body images of the type specimen, the lack of a male specimen has prevented the species being formally redescribed or revised. Fortunately, we have been able to collect several specimens from the type locality in Northeast China of *Kolla albescens*. Based on colour, external features and the male genitalia, this species should be included in the leafhopper genus *Pagaronia* Ball (Evacanthinae: Pagaroniini). Subsequent comparison with figures of *Pagaronia* in Kwon (1981) indicated that it was a senior synonym of *Pagaronia continentalis* Anufriev (1970), the only species of *Pagaronia* from China (Kwon and Huh 2001). Habitus photographs of the male and female and illustrations of the male and female genitalia of *Pagaronia albescens* are provided.

## Material and methods

The male and female genital structures were prepared according to the techniques described by Oman (1949) and Mejdalani (1998), respectively. The dissected parts are stored in small vials with glycerin and attached below the specimens. The morphological terminology adopted herein follows mainly Young (1986), except for the facial areas of the head (Hamilton 1981; Mejdalani 1998), the leg chaetotaxy (Rakitov 1998) and that of the female genitalia (Nielson 1965; Davis 1975; Mejdalani 1998). All specimens studied are housed in the Institute of Entomology, Guizhou University, Guiyang, China (GUGC).

## Taxonomy

### Pagaronia albescens

Taxon classificationAnimaliaHemipteraCicadellidae

(Jacobi, 1943) comb. n.

Figs 1 – 21

Kolla albescens Jacobi, 1943: 28; Wilson et al. (2009a, b)
Pagaronia continentalis Anufriev, 1970, 18: 555; Kwon 1983: 18 (in key). **syn. n.**

#### Type-locality.

“Gaolinzsy” (NE China).

#### Description.

Length of males 8.5–9.0 mm, females 8.7–9.8 mm.

Coloration. Male: Head, thorax and pygofer yellowish-white; apical 1/3 of crown with three transverse black spots, one at median portion and one at each lateral margin; forewing with brown costal margin; abdomen orange-yellow in ventral view, pygofer yellow-white. Female: Head, thorax and abdominal sternites yellowish-brown; forewing pale lacteous; other coloration similar to male.

Head anterior margin almost angulate; median length of crown 5/7 of interocular width; coronal suture distinct at posterior half of crown; crown concave between ocellus and coronal suture, with fovea between ocellus and eye; ocelli located in front of imaginary line between anterior eye angles, each closer to eye than to median line of crown; lateral frontal sutures extending onto crown, attaining ocelli; frons flattened medially, muscle impressions distinct in female and obscure in male; epistomal suture complete. Pronotum broader than head; anterior margin broadly rounded; posterior margin concave medially; disk transversely rugulose medially; dorsopleural carinae distinct; scutellum flattened behind transverse depression; transverse depression straight, located in middle of disk, attaining lateral margins of scutellum; forewing (Fig. 12) with base of second apical cell more proximal than base of third; medial inner subapical cell open. Fore legs with femur (Fig. 13), in anterior view, with intercalary row comprising about 10 widely spaced stout setae on distal half, first anteromedial seta (AM1) located near base of ventroapical femoral lobe, anteroventral row with about four stout setae. Hindleg with apical femoral setal formula 2:1:1. Male abdominal apodemes small, reaching near mid length of third segment.

**Figures 1–6.**
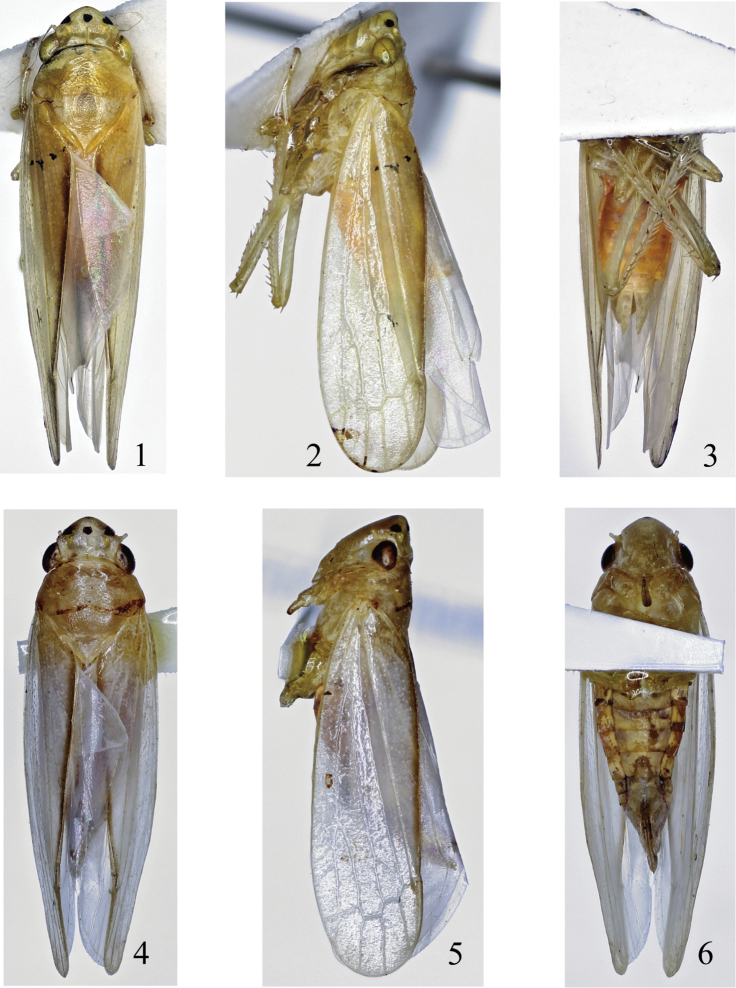
*Pagaronia albescens* (Jacobi, 1943), comb. n. **1–3** body of male (9.0 mm): **1** dorsal view **2** lateral view **3** ventral view **4–6** body of female (9.8 mm) **4** dorsal view **5** lateral view **6** ventral view.

**Figures 7–13.**
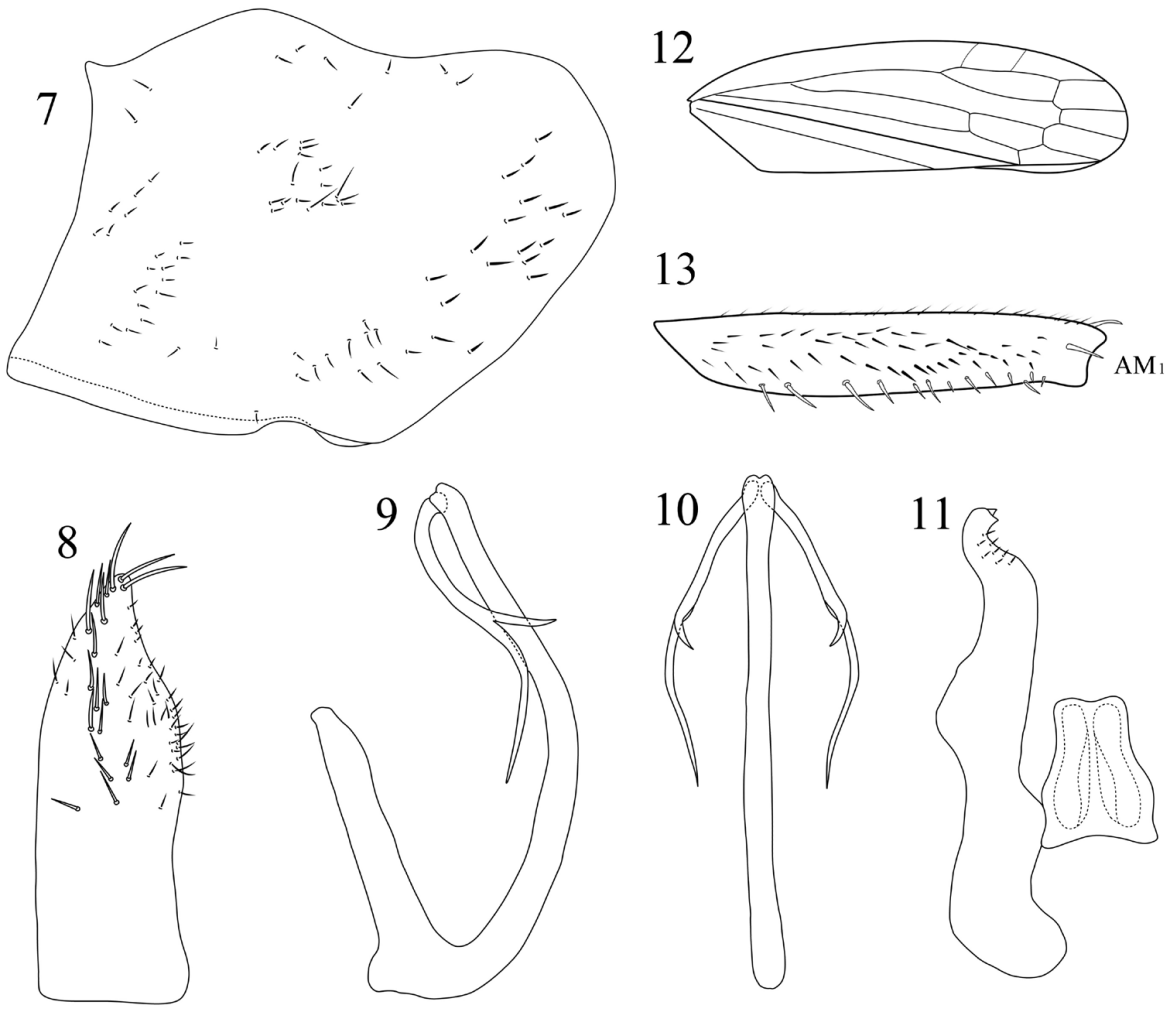
*Pagaronia albescens* (Jacobi, 1943), comb. n., male genitalia **7** pygofer, lateral view **8** subgenital plate, ventral view **9** aedeagus, lateral view **10** aedeagus, caudoventral view **11** connective and style, dorsal view **12** forewing **13** fore femur, anterior view. AM_1_ = first anteromedial seta.

Male genitalia with pygofer (Fig. 7), in lateral view, broad and strongly produced posteriorly, dorsal margin with anterior half convex and posterior half slightly concave; posterior margin broadly rounded; ventral margin with fold, fold concave near apex; disk apicoventral portion with sparse short macrosetae; microsetae also present. Subgenital plate (Fig. 8) small, distinctly shorter than pygofer; posterior area abruptly narrowed, apex nearly acute and slightly curved outwards; apical half with sparse microsetae and irregularly triseriate macrosetae medially. Aedeagus (Figs 9, 10) with long basal apodeme, as long as half of shaft; aedeagal shaft slender, curved posterodorsally, with apical aedeagal processes branched, longer branch extending beyond mid-length of shaft with short sub-branch medially. Connective shield-shaped (Fig. 11), with basal half broader. Style (Fig. 11) strong and extending posteriorly beyond connective apex, preapical portion with several microsetae; apical portion curved, S-shaped, and apex with two denticles.

**Figures 14–21.**
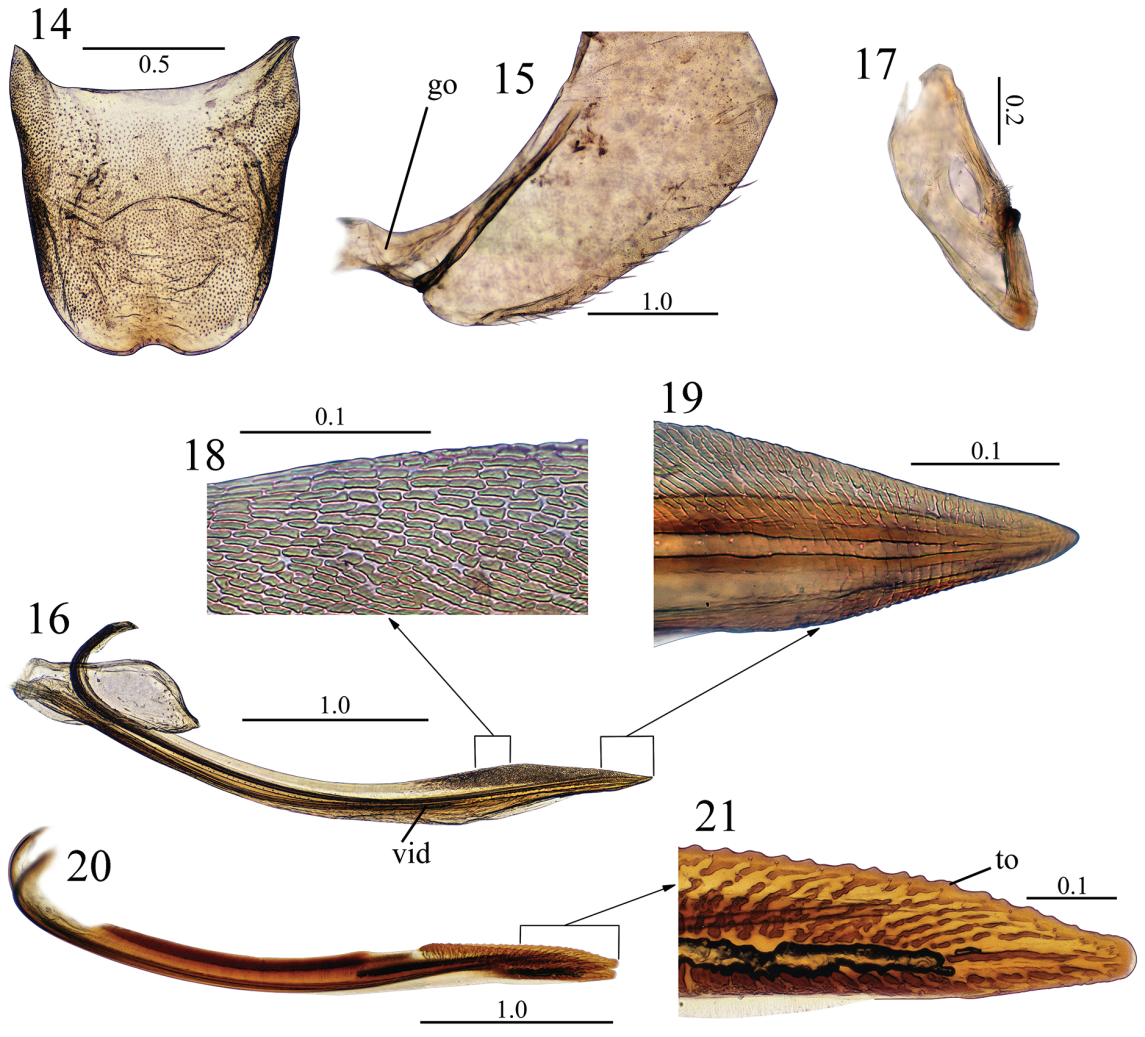
*Pagaronia albescens* (Jacobi, 1943), comb. n., female genitalia: **14** sternite VII, ventral view **15** pygofer, lateral view **16** valvifer I and valvula I, lateral view **17** valvifer II, lateral view **18** dorsal sculptured area of valvula I, lateral view **19** apical portion of valvula I, lateral view **20** valvulae II, lateral view **21** apex and apical portion of valvulae II, lateral view. go = gonangulum, to = tooth, vid = ventral interlocking device. Scale bars in millimeters.

Female genitalia. Sternite VII (Fig. 14), in ventral view, slightly longer than broad; posterior margin convex and with shallow concavity medially; surface with few small setae mostly on anterior half. Pygofer (Fig. 15), in lateral view, moderately produced posteriorly; posterior margin with subacute apex, dorsoposterior margin obliquely truncate; surface with macrosetae mostly on ventral margin, arranged almost in a row. Valvifers I (Fig. 16), in lateral view, longer than tall; posteroventral margin angulate. Valvifers II (Fig. 17), in lateral view, nearly fusiform, with small group of clustered setae near articulation point, articulation point located on 2/3 of dorsal margin. Valvulae relatively narrow in lateral view. Valvulae I (Fig. 16) with base subtriangular in ventral view; with convex lateral outer margin; in lateral view (Figs 16, 18, 19) with dorsal and ventral margins nearly parallel over basal two thirds behind basal curvature, thereafter slightly convex and narrowed to acute apex; dorsal sculptured area restricted to posterior 2/3 of shaft, formed mostly by subrectangular sculpture arranged in oblique lines, except basally were it is arranged horizontally; ventral sculptured area formed by dense imbricate sculpture restricted to apical portion of shaft; length of ventral interlocking device corresponding to approximately 2/3 of blade length beyond basal curvature. Valvulae II (Figs 20, 21), in lateral view, with anterior fused basal section nearly 2/3 length of blade; only slightly expanded beyond basal curvature and dorsal hyaline region; dorsal and ventral margins approximately parallel; apex narrowly rounded; preapical prominence absent; shaft bearing approximately 25 teeth (Fig. 21: to) over posterior 1/3 of blade; each tooth subtriangular; apicoventral margin without distinct teeth; teeth and dorsal margin of shaft without secondary denticles; ducts sparse, extending toward teeth and toward apical blade portion. Gonoplacs, in lateral view, expanded at apical half; apex obtuse; surface with macrosetae mostly distributed on apical portion and extending anteriorly along ventral margin of apical half.

#### Known distribution.

Russia, Korea, China.

#### Material examined.

3 males and 5 females, China, Liaoning Province, Henren County, Benxi Laotuding Preserve, 19 to 21 July 2011, coll. Fan Zhi-hua and Yu Xiao-fei; 1 female, China, Jilin Province, Mt. Changbai, 24 July 2011, coll. Yu Xiao-fei.

#### Remarks.

This species was described from a single female specimen (holotype) from “Gaolinzsy” (NE China). The type specimen, deposited in Deutsches Entomologisches Institut im ZALF, Müncheburg, Germany, has not been examined but our material has been compared with an image of the type (see introduction). The identity and reference for the *Pagaronia* species recorded from China in Hayashi et al. (2010) and Kamitani et al. (2012) was established as *Pagaronia continentalis* recorded by Kwon and Huh (2001) (M. Hayashi, pers. com.).

## Supplementary Material

XML Treatment for Pagaronia albescens
